# Phage Therapy: What Have We Learned?

**DOI:** 10.3390/v10060288

**Published:** 2018-05-28

**Authors:** Andrzej Górski, Ryszard Międzybrodzki, Małgorzata Łobocka, Aleksandra Głowacka-Rutkowska, Agnieszka Bednarek, Jan Borysowski, Ewa Jończyk-Matysiak, Marzanna Łusiak-Szelachowska, Beata Weber-Dąbrowska, Natalia Bagińska, Sławomir Letkiewicz, Krystyna Dąbrowska, Jacques Scheres

**Affiliations:** 1 Bacteriophage Laboratory, Ludwik Hirszfeld Institute of Immunology and Experimental Therapy, Polish Academy of Sciences, Rudolfa Weigla Street 12, 53-114 Wroclaw, Poland; mbrodzki@iitd.pan.wroc.pl (R.M.); ewa.jonczyk@iitd.pan.wroc.pl (E.J.-M.); marzena@iitd.pan.wroc.pl (M.Ł.-S.); weber@iitd.pan.wroc.pl (B.W.-D.); natalia.baginska@iitd.pan.wroc.pl (N.B.); dabrok@iitd.pan.wroc.pl (K.D.); 2 Phage Therapy Unit, Ludwik Hirszfeld Institute of Immunology and Experimental Therapy, Polish Academy of Sciences, Rudolfa Weigla Street 12, 53-114 Wroclaw, Poland; letkiewicz1@o2.pl; 3 Department of Clinical Immunology, Transplantation Institute, Medical University of Warsaw, Nowogrodzka Street 59, 02-006 Warsaw, Poland; jborysowski@interia.pl; 4 Institute of Biochemistry and Biophysics, Polish Academy of Sciences, Pawińskiego Street 5 A, 02-106 Warsaw, Poland; lobocka@ibb.waw.pl (M.Ł.); glowacka@ibb.waw.pl (A.G.-R.); a.kielan@ibb.waw.pl (A.B.); 5 Autonomous Department of Microbial Biology, Faculty of Agriculture and Biology, Warsaw University of Life Sciences, Nowoursynowska Street 159, 02-776 Warsaw, Poland; 6 Medical Sciences Institute, Katowice School of Economics, Harcerzy Września Street 3, 40-659 Katowice, Poland; 7 Research and Development Center, Regional Specialized Hospital, Kamieńskiego 73a, 51-124 Wrocław, Poland; 8 National Institute of Public Health NIZP, Chocimska Street 24, 00-971 Warsaw, Poland; jscheres@icloud.com

**Keywords:** phage therapy, experimental therapy, phage cocktails, anti-phage antibodies, prophage, immunomodulation

## Abstract

In this article we explain how current events in the field of phage therapy may positively influence its future development. We discuss the shift in position of the authorities, academia, media, non-governmental organizations, regulatory agencies, patients, and doctors which could enable further advances in the research and application of the therapy. In addition, we discuss methods to obtain optimal phage preparations and suggest the potential of novel applications of phage therapy extending beyond its anti-bacterial action.

The intention of this article is to highlight the current events and issues related to phage therapy (PT) which seem to be most relevant for its further progress. These issues correspond to two main topics addressed in our article: the regulatory/ethical/awareness raising topic, which will subsequently yield to the topic of lysogeny/immunity/optimal use of phage preparations. These issues appear to be especially timely and relevant from the perspective of our team with leading expertise in PT among the EU countries. 

## 1. More Room for Phage Therapy on the Horizon?

After decades of being kept out of the mainstream infectious disease armamentarium of the Western world, there now appears to be a silver lining on the horizon for phage therapy. PT is shedding its dubious associations with alternative and fringe medicine. Triggered by the growing threat of antibiotic resistance, there is a slow but substantial change in the appreciation of PT and a more permissive attitude of the main stakeholders in the infectious disease arena. Reviews on PT covered by PubMed appear almost every month. According to Web of Science, their average citation number per annum in recent years has been around 1100, and increased to approximately 1400 in 2017. There is also a growing understanding of the ethical, legal, and administrative rules relevant to experimental therapy which currently allow such treatment to be provided to patients for whom all other available therapies have failed. Below are some observations and reflections on the present attitudes of doctors, patients, academia, policymakers, media, and industry towards PT.

## 2. Doctors, Pharmacists, and Academia

The professionals in the fight against serious infections are doctors, general practitioners, infectiologists or medical microbiologists, and pharmacists. In the case of a serious infection, they have to choose the most appropriate remedy. From the plenitude of available antibiotics, they select those for which the pathogen in question tests sensitive, and standard application protocols are followed. However, almost every day doctors and pharmacists are confronted with pathogens that are increasingly resistant to certain or even a long list of antibiotics. More and more they feel the urgent need for new antibiotics or other instruments to help them improve or even save the lives of their critically ill patients. Without effective antibiotics (and thus effectively standing helpless), doctors eagerly look for alternatives. Phage therapy might represent such an alternative, at least in certain cases. In the last decade, many publications on bacteriophages and their possible applicability have appeared regularly in the clinical, applied, and fundamental scientific microbiological literature [1,2,3]. PT is often a specific subject on the programme of clinical and fundamental microbiological conferences, and is sometimes even the sole focus of dedicated PT symposia. As a result, a growing group of physicians and pharmacists in Western countries are acquainted with the potency and the pros and cons of PT as a possible alternative or an auxiliary therapy in cases of untreatable antibiotic-resistant infections, which is applied in neighbouring non-EU countries on the continent. Publications of successful and sometimes spectacular phage therapy cases trigger this interest, and in their aftermath often lead to a flow of requests by doctors to phage laboratories for help in analogous cases. Such requests keep coming, even from places where phages are not officially registered medical products, and PT still is generally not available in most of the West, although it is sometimes available experimentally. Therefore, in some cases doctors refer or mediate their patients to recognized PT centres elsewhere, such as in Poland (Wroclaw) and Georgia (Tbilisi). 

In general, Western medical professionals show signs of increased openness towards PT as a possibly valuable additional tool in the fight against resistant and seriously threatening or disabling infections. At the same time, quite a long road of broad basic research, robust clinical trials, adjustment of the regulatory systems, education, and training still lies ahead before PT becomes practical, optimally effective, and compatible with the rules. Nevertheless, it may be advisable for doctors and medical students, pharmacists and pharmaceutical students to inform themselves in anticipation of the possible role of bacteriophages in infectious disease treatment. Is it not amazing that most medical professionals do not know about bacteriophages, these evolutionarily important creatures which are at least ten times more frequent in the microbiome than all bacteria and also greatly outnumber them within our body?

In this field, certain non-governmental organizations (NGOs) of professionals sometimes arise and intend to fulfil a role in closing the existing knowledge gap and building the bridge to formal recognition of PT. For instance, P.H.A.G.E. (Phages for Human Applications Europe Group) is a multidisciplinary group of doctors with practical experience or strong interest in PT, basic and applied phage researchers, and policymakers [4]. The exchange of phages, knowledge, and technology, participation in projects, organizing conferences and presentations all over Europe, publication, and education are among its main activities.

In 2015, a number of attendants of a bacteriophage conference in Tbilisi (Georgia) composed a multidisciplinary and intercontinental expert panel to establish an academic and medical initiative for the re-implementation of PT. The papers on the “Silk Route to the acceptance and re-implementation of bacteriophage therapy” which have recently been produced by this expert round-table are a significant contribution to the development of international guidelines and frameworks which are needed for a legal and effective application of bacteriophage therapy by physicians and the receiving patients [5,6].

Phages for Global Health is another very interesting multidisciplinary organization. Its mission is “to bring phage expertise to the developing world”. Developing countries are disproportionally impacted by infectious diseases (e.g., *Campylobacter* infection has a fatality rate of about 0.1% in wealthy countries, but 8.8% in Kenya, mostly children) [7]. Phages for Global Health provides laboratory training workshops, teaching phage biology to scientists on location in developing countries where the need for alternatives to antibiotics (e.g., PT) is felt especially [8]. In addition, product development projects are performed in which international multidisciplinary teams are built that co-develop phage products for specific applications in developing countries [9]. In June and July 2018, the Second East African Phage Workshop will be held at Pwani University in Kilifi, Kenya. The participants will learn how to isolate and characterize phages as antibiotic alternatives for use against antibiotic-resistant bacteria. 

## 3. CRISPR-Cas: From Phages to Eukaryotes

An additional important referral should be made to the recent development of simplified methods for high-efficiency gene-editing. This spectacular innovative technology is based on the CRISPR-Cas mechanisms which bacteria developed during their evolution in order to protect themselves against infections by phages. This has once again made clear how interesting and important the study of the very old relationship between phages and bacteria can be, and that it can lead to unexpected benefits and great leaps forward for science and its practical applications, including great promises for the prevention or treatment of genetic and complex diseases [10,11,12].

## 4. Patients, the Media, and PT

The patient, not the doctor, is the primary stakeholder in health and health care. Stimulating patient empowerment, health literacy, shared decision-making, and personal responsibility are core elements of health policy in almost all countries. Especially when the doctors can neither heal nor help with the existing medical means (e.g., in cases of incurable cancer), it is often the patient who opens the question of alternative therapies and asks for a referral to any other centre that might be able to help them, wherever on Earth, with whatever therapy, and at whatever costs. Sometimes the patient or their relatives are, via the internet, well-informed about possible alternatives. Asking for a second opinion has become the generally accepted standard. This pattern also applies to phage therapy. Though still quite exceptional, there are patients with chronic untreatable threatening resistant infections who indeed know about the option of bacteriophages, and ask their doctor to try phage therapy or to refer them for it. A growing number of patients find their way to bacteriophage centres abroad, staying there several weeks for phage selection and initial therapy, and are willing to bear the total costs of treatment, travel, and accommodation themselves. This medical tourism for phage therapy has grown especially since the media have taken their own responsibility in the national campaigns against the inappropriate use of antibiotics and have also informed the general public about PT as an alternative. They often mention PT as being applied in Central and Eastern European countries, and have reported spectacular cases of wound healing and the prevention of diabetic limb amputations with phages. Phage stories with basic information and successful cases of PT including the places where and how you can access it appear on TV [13,14] and in a broad range of societal magazines, ranging from knowledge magazines such as Der Spiegel Wissen in Germany [15] or Elsevier Weekblad [16] to the popular women’s magazine Libelle [17] in the Netherlands. So, thanks to some pioneering patients and with the help of the media, PT has gained a place on the stage for the general public in the Western world—almost a century later than in the East.

## 5. Industry and SMEs

To make its way from the experimental level towards registration for safe application in human medicine, PT needs the engagement of a dedicated industry which is willing to produce phages following the safety and quality requirements [18], requiring high investments. So far, very few firms, usually SMEs, have chosen to engage in the production of phages ready for use in clinical trials and human application, usually in the context of developmental projects performed in cooperation with research institutes, academia, and or state laboratories. This contrasts somewhat with the food, disinfection, cosmetic, and veterinary sector, where phages and phage products (lysins) have already reached consumers. The US Food and Drug Administration (FDA) has approved a small number of products for these markets, and several applications are in the pipeline for approval. Very recently, the phage-producing SME, Phage Technology Center GmbH [19], was present at the international Anuga FoodTec International Food Technology Fair (Cologne, Germany, March 2018), presenting its phages against *Salmonella* and *E. coli* for various food applications. According to its Senior Manager Research & Development, the market for phages is going to boom in this sector, which is certainly not yet the case in human medicine.

## 6. Authorities and PT

Globally, national authorities consider antibiotic resistance to be a profound threat to health. Their national strategies, action plans, and preventive campaigns focus on a more appropriate use of antibiotics and the search for alternatives. The development of vaccines, innovative diagnostic tests, and novel interventions are usually mentioned as alternatives. Only very exceptionally are the words bacteriophage, PT, or phage products (lysins or endolysins) found in the action plans. The main reason is the current lack of positive clinical trials with PT. The reputation and successes of PT in countries with longstanding application of PT are distrusted and considered to be poorly documented, not convincing and not proven, and serious adverse effects of PT are feared or at least not to be excluded. The dictum *primum non nocere* (first do not harm) and quality assurance, both based on solid clinical trials according to the standard rules, are indeed strong pillars of drug policy. For similar reasons, there is no mention of PT in the five-year action plan of the European Commission against antibiotic resistance launched in 2012 and updated in 2017 [20]. The words “phage therapy” and “phages” are also lacking in the global action plan to tackle antimicrobial resistance which was endorsed in May 2015 by the World Health Assembly in Geneva [21]. This action of the Assembly was truly a unique one, showing the United Nations’ serious concern that antibiotic resistance “threatens the very core of modern medicine and the sustainability of an effective, global public health response to the enduring threat from infectious diseases”. Is this emergency situation still not serious enough to allow a little more place for phage therapy, a method which a century ago was effectively applied and appreciated in curative care and public health, in the East and in the West before we used antibiotics? 

Fortunately, the position of the authorities appears to be shifting, albeit slowly, towards more latitude for phage therapy. This may be illustrated by the following selection of interesting formal actions and documents of authorities in the US and/or the EU:

In mid-2017, the U.S. Food and Drug Agency (FDA), the National Institutes of Health, the National Institute of Allergy and Infectious Diseases, and the Center for Biologics Evaluation and Research co-organized a two-day workshop to facilitate the development of a rigorous clinical assessment of bacteriophage therapy [22].

In late 2017, the FDA also gave the status of Emergency Investigational New Drug to phages specifically active against a multidrug-resistant *Acinetobacter baumannii*, which were applied in a patient with septic shock who improved within days and survived, being the first case of intravenous use for systemic infection. The phages were obtained from the US Navy and Texas A&M University, in combination with the San Diego biotech firm AmpliPhi [23].

Later, the FDA gave its seal of approval to a new phase I/II clinical trial in humans at Mount Sinai Hospital in New York City to test a new bacteriophage treatment for Crohn’s disease [24]. 

In 2013, the European Commission funded the PHAGOBURN project co-ordinated by the French Ministry of Defence, with partners from France, Belgium, and Switzerland. The main objective of the project is “to assess the safety, effectiveness and pharmacodynamics of two therapeutic phage cocktails to treat either *E. coli* or *P. aeruginosa* burn wound infections” [25].

In 2015, the White House National Action Plan for combating Antibiotic-Resistant Bacteria launched by the White House in 2015 listed “the use of phage and phage derived lysins to kill specific bacteria while preserving the microbiota” among the non-traditional therapeutics which should be further developed [26].

The Transatlantic Taskforce on Antimicrobial Resistance (TATFAR) was created in 2009 to enhance synergy and communication between government agencies on both sides of the Atlantic Ocean. The first partners were the US and the EU, and Canada and Norway joined later . Action no. 3.6 of TATFAR’s updated action plan is: “Exchange information on possible regulatory approaches to development of alternative approaches for managing bacterial infections, such as bacteriophage therapy and vaccines for health care associated infections (joint action by FDA, EMA, HC, and NMA)”[27]. In a message from the recent TATFAR meeting (Atlanta CDC 7–9 of March 2018), according to Marco Cavaleri from EMA (personal communication, March 12, 2018) it was reiterated that in the discussion on the alternatives to antibiotics, phages should be on the radar as an option that deserves to be discussed across the Atlantic. The biggest problem is that not many companies are interested in discussing the topic or in considering how to approach clinical development.

In its German Antimicrobial Resistance Strategy entitled “DART 2020: Fighting antibiotic resistance for the good of both humans and animals”, the Federal Ministry of Food and Agriculture announced plans to assess the “Possible positive effects of bacteriophages and other substances to reduce or eliminate bacteria on carcasses as a supplement to process hygiene”. Though this action is clearly meant to improve food hygiene and not as phage therapy for human patients, it is nevertheless noted here because it is one of the very few governmental documents mentioning bacteriophages as a means to fight antibiotic resistance, for the good of humanity and animals [28].

The same action point was proposed by the Federal Government of Germany in the report “Combating Antimicrobial Resistance. Examples of Best-Practices of the G7 countries” of the G7 GERMANY 2015 meeting in Berlin [29].

Very recently, a major, hope-giving and possibly historical step for the applicability of PT was taken by the Belgian Federal Government (January 2018) [30]. In cooperation with academia (including ethicists), researchers and experts from the care sector the Federal Agency for Medicines and Health Products succeeded in developing a regulation for phage production and the clinical application of PT. The procedure, which obtained its legal approval at the end of January 2018, is based on the legal possibilities in Belgium for a pharmacist to prepare a medical product (including phages) for an individual patient. The active ingredients used in this so-called magistral preparation (in the US, “compound prescription drug preparation”) must meet the requirements of the European, Belgian, or another official Pharmacopoeia. If this magistral route would be copied *mutatis mutandis* by other countries, it would truly represent a breakthrough for the application of PT, especially in individual life-threatening situations (based on the Declaration of Helsinki, WMA, 1964) [31]. In fact, a similar approach has long been in use in Poland at the Phage Therapy Center of the Institute of Immunology and Experimental Therapy [32,33].

## 7. National Regulations Enabling Experimental Therapy (Including PT)

In view of these developments, it might be useful to summarize the current status of experimental therapy in Europe and elsewhere.

Generally, every medicinal product must be approved by a relevant regulatory agency before it can be used in clinical practice. However, in response to the needs of patients who cannot be treated satisfactorily with authorized drugs, many countries have introduced regulations which enable doctors to use experimental treatments. 

In the European Union (EU), the legal framework for treatment with unauthorized medicinal products (termed compassionate use—CU) was introduced by Article 83 [34] of Regulation (EC) No. 726/2004 of the European Parliament and of the Council. This article permits the use of unauthorized medicinal products in groups of patients, provided that two main requirements are met: (1) the patient has a chronically or seriously debilitating disease, or a life-threatening disease which cannot be treated satisfactorily with an authorized medicinal product; and (2) the medicinal product must be either the subject of an application for a centralized marketing authorization or be undergoing clinical trials. Specific CU programs are to be implemented and governed by individual Member States (MSs) [34]. As of 2016, 18 out of 28 MSs had specific CU regulations and 20 had implemented CU programmes [35]. Moreover, Article 5 of Directive 2001/83/EC of the European Parliament and of the Council allows the use of unauthorized medicinal products in individual patients under the direct responsibility of a healthcare professional (i.e., named-patient basis treatment) [36].

In the US, according to the terminology adopted by the FDA, the use of unauthorized drugs outside of clinical trials is called expanded access (EA). General requirements for EA include the following: (1) a serious or immediately life-threatening disease where no comparable or satisfactory alternative therapy is available; (2) the potential benefits justify the potential risks and the potential risks are not unreasonable in the context of the disease; (3) there is no threat to the initiation, conduct, or completion of clinical trials; (4) informed consent of the patient; (5) Institutional Review Board (IRB) review [37,38]. Independently of the existing FDA regulations, 38 states have recently introduced so-called right-to-try laws which are to facilitate access of terminally ill patients to investigational drugs that have completed phase I of a clinical trial. However, these laws have been heavily criticized by experts for offering “false hope” to patients without providing any actual improvements in access to investigational drugs [39]. Nevertheless, at the time of this writing, US Congress has passed a relevant bill which has been a priority of President Trump [40]. If approved by the US Senate, the law would allow patients to sidestep FDA approval once they have received permission from a company [41].

In Canada, the use of unauthorized drugs is legally permissible in Special Access Programmes (SAPs). Basic information about these programmes is available in the Guidance Document for Industry and Practitioners—Special Access Programme for Drugs developed by the Canadian regulatory agency Health Canada [42]. Under SAP rules, an unauthorized drug can be used in patients with serious or life-threatening diseases, especially in emergency cases when conventional therapies have failed, are unsuitable, or are unavailable. The use of an unauthorized drug must be supported by some credible evidence of its safety and efficacy, and a doctor should obtain informed consent from the patient.

In Australia, there are two schemes that enable doctors to use unauthorized drugs: the Authorized Prescriber Scheme (APS) and the Special Access Scheme (SAS) [43]. In APS, an application for the use of an unauthorized drug needs to be approved by a bioethics committee or endorsed by a specialist in a discipline relevant to the proposed treatment. Important issues that are evaluated include the qualifications and experiences of the doctor, access to facilities necessary to perform the treatment, evidence to support the proposed treatment, clinical justification including whether other therapeutic alternatives have been tried, and an explanation of why the unauthorized drug is proposed. In addition, informed consent of the patient is required. Under this scheme, the doctor can be granted permission to prescribe a specified unauthorized drug to specific patients (or groups of patients) with a particular disease. In the other major Australian scheme (the SAS), unauthorized drugs can be used in single patients on a case-by-case basis. It is expected that before use of an unauthorized drug, all authorized treatment options will be considered. The doctor must also obtain informed consent from the patient. Moreover, in cases when the treated disease is not life-threatening and the unauthorized drug does not have an established history of use, clinical justification for the use of an unauthorized drug must also be provided.

## 8. Important Issues Which Need Addressing to Enable Further Progress and Optimization of PT and Relevant Clinical Trials

In this part of our article, we wish to briefly discuss the issues pertinent to PT that have not been dealt with adequately so far, and where the advancement of our knowledge may lead to a faster introduction of phages to the health market.

The long-lasting effects of PT confirm its safety. Even though the therapeutic value of PT still awaits confirmation by clinical trials—in line with the requirements of evidence-based medicine—as pointed out by a former FDA commissioner: “Although randomized trials perform an essential role in the development of therapies, we should not neglect the crucial and complementary role than can be played by high-quality observational studies” [44]. In this regard, our results of suggested PT efficacy appear to be quite encouraging (>50% success rate using purified phage preparations), while the safety of the therapy is remarkable [32]. This has been confirmed by our recent preliminary analysis of remote observations in a group of 33 patients who completed PT up to 7 years ago. When questioned, two-thirds of those patients were satisfied with therapy results and, importantly, none of them reported any complications that could be related to PT [45].

## 9. PT and Antibody Responses against Phages

Our studies in animals and patients have provided interesting and potentially useful information on anti-phage antibody responses during PT. Among healthy donors, 29–82% may be positive for serum anti-phage antibodies depending on phage type (anti-T4 coliphage antibodies being most common) [46]. Antibody responses during PT have been described by us in detail. In patients awaiting PT, very low levels of anti-phage antibodies were detectable (mean K index in 60 patients was 0.17), while the index could reach values as high as 200 during PT. Furthermore, purified phage preparations seem to induce higher antibody responses than do the lysates. In addition, identical phages can elicit different levels of antibody responses in patients, which may depend on the immune reactivity of those patients. The most important finding has been that a good clinical outcome of PT may be observed in patients with high antibody responses [47]. Our recent analysis suggests that there is an association between the duration of therapy and antibody responses (for *Staphylococcus* phages, the Spearman correlation was 0.856, *p* < 0.0001). Similar data were obtained in mice [46]. While high antibody responses do not appear to affect the outcome of PT, we prefer to terminate the therapy if the antibody levels are high to avoid possible complications in the future (e.g., the unknown effect of phage–antibody complexes).

## 10. Monotherapy vs. Phage Cocktails

The issue of phage cocktails vs. monovalent phage preparations remains undecided: our preliminary data might suggest that there is no significant difference in the therapeutic efficacy between these preparations, while the frequency of high antibody responses was higher in patients treated with cocktails compared to those on monotherapy [48].

## 11. Optimal Clinical Models for PT and Prognosis of Therapy

One of the key questions asked by the Guest Editor of this volume, Prof. H. Brüssow, was: is it possible to formulate a set of rules with respect to infection type, which predict successful interventions? [49] Our experience so far suggests that intrarectal PT of chronic bacterial prostatitis offers the highest success rate [50]. Several factors could be responsible for those results, among them possible good penetration of phages from the rectum to the prostatic tissue (phage ability to penetrate cell layers has recently been demonstrated) [51,52], eradication of rectal carriage of a pathogen, as well as low anti-phage antibody responses elicited by this mode of phage administration [47]. Our data on patients’ immunomonitoring suggest that an increase in phagocytosis may be a good prognostic sign of PT success [53].

## 12. Mouse Model of Acute Urinary Tract Infection Confirms Neutrophil–Phage Synergy

The value of this parameter has been confirmed by an experimental study in mice. The experiments were performed on a mouse model of acute urinary tract infection [54] caused by transurethral bacterial inoculation with uropathogenic strain isolated from patients: *E. faecalis* 15/P or *P. aeruginosa* 119×. Spleen mononuclear cells were isolated according to the method described by Kruisbeck (2000) [55] using a density gradient (Histopaque-1083, Sigma-Aldrich, St. Louis, MO, USA). Intracellular killing of bacteria by splenic macrophages was tested according to the method described by Buisman et al. (1991) and Leijh et al. (1982) [56,57]. The obtained value corresponded to the percentage of killed phagocytosed bacteria, and it was examined both 3 and 6 days after the infection. In the infected group of DBA1/LAC J mice (*n* = 6) (without phage treatment), significantly lower (Mann–Whitney U-test, *p* = 0.004) intracellular killing of a pathogenic bacterial strain (the same as the cause of infection) by splenic mononuclear cells (63.2% ± 7.1 for mice infected with (*P. aeruginosa*) was observed when compared to the bactericidal capacity of healthy animals (82.8% ± 8.0). Reduced intracellular killing was observed in infected mice on days 3 and 6 after the infection, regardless of the uropathogenic strain used. Importantly, the intraperitoneal administration of the phage lysate (at a concentration of 5 × 10^10^ pfu/mL) exerted a stimulatory effect on the spleen phagocytes in the group of mice with experimentally-induced infection by *E. faecalis* 6 days after sequential application of three doses (1 h, 24 h, and 48 h after bacterial inoculation) of specific enterococcal phage lysate Ent 15/P (86.7% ± 3.8) when compared to non-treated mice (74.1% ± 9.2) (Mann–Whitney U-test, *p* = 0.014). An improvement in bactericidal activity of splenic mononuclear cells was also obtained for a group of mice treated with three doses of the phage lysate (86.7% ± 3.8) after 6 days of infection when compared to the same group tested 3 days after bacterial inoculation (72.2% ± 6.7, Mann–Whitney U-test, *p* = 0.004). The improvement of splenic macrophage anti-bacterial function was paralleled by a significant fall of bacteria counts in liver, kidneys, and urinary bladder of phage-treated mice [58]. Recent data fully confirm this assumption by showing that neutrophil–phage synergy is needed for successful PT of experimental pneumonia in mice [59].

## 13. Prophages in Bacterial Strains Used for Therapeutic Phage Propagation: Their Significance, Detection, and Elimination 

### Bacterial Strains for the Propagation of Therapeutic Phages 

Sources of phages for therapeutic use are lysates of cells that serve for the propagation of those phages. In addition to the desired phage, they contain bacterial cell components and may contain contaminating phages that are produced as a result of prophage induction if the phage propagation strain is a lysogen [60,61]. Genome analysis of bacterial strains used for phage propagation reveals not only genes that encode toxins or other virulence determinants, but also mobile genetic elements, including plasmids, transposons, and prophages. The presence of toxins in lysates increases the cost of lysate purification. The presence of mobile genetic elements poses a risk of uncontrolled spread of bacterial virulence or antibiotic-resistance genes. The most problematic lysate contaminants are temperate phages. Due to the physico-chemical similarity of contaminating temperate phages and lytic phages, the former are practically inseparable from the main phage population in a lysate. Despite the possibilities of their detection in lysates and even the estimation of what fraction of the total phage population is represented by them [61], the only way to eliminate them is the construction of phage propagation strains that are depleted of prophages [60,62]. 

A key argument for the removal of active prophages from the genomes of bacteria that serve as therapeutic phage propagation strains is the prophage genetic load. Temperate phages are major driving forces of horizontal gene transfer and bacterial evolution [63,64,65]. They typically carry genes that encode functions which are adaptive for their bacterial hosts, and in that way decrease the probability of overgrowth of the bacterial population by cells that have lost them. In the case of prophages and plasmids of bacterial pathogens, the adaptive functions encoded by these elements are nearly always associated with better adaptation of the bacteria to pathogenicity [63,65,66,67,68,69,70,71,72]. In addition to virulence factors, certain prophages encode homologs of error-prone DNA polymerase *V subunits* [73,74], and were proposed to play roles in the diversification of bacterial strains (e.g., by facilitating the acquisition of resistance to toxins or antimicrobials by mutations) [75]. Temperate phage virions that contaminate therapeutic phage preparations act not only as vectors of their own DNA, but can also act as vectors of bacterial, plasmid, or pathogenicity island DNA [76,77,78,79,80,81,82,83]. For instance, a spontaneous intraspecies transfer of the blaNDM-1 carbapenemase gene from a carbapenem-resistant strain containing two active prophages to a carbapenem-sensitive *Acinetobacter baumannii* strain was attributed to the transduction mediated by a prophage-derived temperate phage [84]. Undoubtedly, the release to the environment of temperate phages containing their own DNA or sometimes even the DNA of plasmids or bacteria derived from contaminated therapeutic phage preparations can contribute to the spread of virulence or antibiotic-resistance genes. In the worst-case scenario, the contaminating phages could be acquired by the infecting bacteria during phage therapy and make these bacteria more pathogenic, negatively influencing the treatment outcome. Although incidents of adverse effects of phage therapy have been surprisingly rare, the possibility of such a scenario should be taken into consideration and avoided when possible, especially in view of the emergence of strains resistant to certain therapeutic phages in the course of phage therapy [32].

Although only about a half of the sequenced bacteria are lysogens, prophages are more frequent in pathogens [85,86,87]. Their abundance varies among different species of pathogenic bacteria. However, bacteria that are known for especially good adaptation to pathogenicity and for their fast acquisition of antibiotic resistance, including ESKAPE pathogens (*Escherichia coli*, *Staphylococcus aureus*, *Klebsiella pneumoniae*, *Acinetobacter baumannii*, *Pseudomonas aeruginosa,* and *Enterococcus* spp.), are often or even in most cases polylysogens [86,88,89,90,91,92,93,94,95,96,97,98,99,100,101,102,103]. Active and defective prophages in the genomes of certain pathogenic bacterial strains (e.g., *E. coli* O157:H7 strain Sakai, or highly virulent *S. pyogenes* strain MGAS315) can occupy as much as about 15% of total genomic DNA [104,105]. 

The ubiquity of lysogeny among bacterial pathogens makes the selection of non-lysogenic bacteria for phage propagation from environmental samples either difficult or impossible. Hence, the identification of active prophages in the genomes of efficient phage propagation strains and their subsequent removal is a strategy of choice in ensuring the monoclonality and safety of therapeutic phage preparations, as well as in decreasing the cost of their production and the evaluation of their purity [60]. 

Prophage-free strains may be acquired from among natural isolates of a given bacterial species or selected from laboratory cultures of prophage-carrying phage propagation strains upon the induction of prophage lytic development and the selection of surviving cells, as reviewed by [60]. Which of these strategies may be optimal depends on several factors. The task may not be simple, as a propagation strain should have all the features of the target bacteria that allow a phage released from this strain to infect the target pathogenic bacterial strain efficiently. 

The stability of lysogeny is associated with numerous factors. In general, the rate of prophage loss by induction increases under conditions of decreased host viability, such as upon exposure to UV, reactive oxygen species, or other mutagenic factors that trigger the SOS response (for review see [106,107,108,109,110,111,112,113]), under high temperatures [114], as well as in the response to certain bacteriocins [115], certain antibiotics that block the action of essential enzymes [93,116] or interfere with intracellular regulatory processes [117,118] or to quorum-sensing signalling molecules [119,120,121]. Typically, induction also occurs spontaneously in a variable fraction of a population of cells [122,123,124,125,126,127,128,129], being responsible for the presence of relevant free temperate phages in the cultures of lysogens [62,130,131]. Thus, derivatives of lysogens that are depleted of certain prophages are expected to occur in nature and in laboratory cultures, although their number may be low, as together with the prophage they lose the prophage-mediated immunity to the infection by the relevant phage. 

## 14. Prophage Detection Methods

Several bioinformatic methods have been developed to identify prophages in bacterial genomes. Programs that implement them can be downloaded from internet resources or are accessible online (e.g., PHAST, PHASTER and PHASTEST [132,133]; Prophinder [134]; Phage_Finder [105]; Prophage Finder [135]; PhiSpy [136]; VirSorter [137]). Their performance is in the range 64–85% for sensitivity and 74–93% for precision when tested with known prophage sequences in complete bacterial genomes [137]. Prophages in the phage propagation strain of a known sequence can also be identified by comparing the sequence of this strain with sequences of other species representatives and by the identification of genome regions that are interrupted by insertions of prophage-size elements [60]. Prophages in the genomes of *S. aureus* or *Salmonella enterica* serovar Typhimurium can be detected by the analysis of PCR reaction products with total genomic DNA of these bacteria and pairs of primers complementary to the conserved DNA regions of their species-specific prophages [94,138,139,140]. The main disadvantage of the aforementioned methods is the distinction of active and defective prophages, which is not always accurate. While defective prophages may be a source of toxins or virulence factors, they are unable to contaminate therapeutic phage preparations in a phage form unless their DNA is not packed into capsids of other phages. To detect active prophages, one should design pairs of primers complementary to the prophage sequences identified in a given strain and use them to amplify the relevant temperate phage DNA with the total virion DNA of a lysate as a template. In our hands, this method works sufficiently well to quickly distinguish active prophages from prophages that cannot produce viable progeny [62]. A necessary condition is to degrade host DNA in a lysate prior to the amplification experiments. 

The sensitivity of contaminating phage detection may be increased by inducing prophage lytic development, with the most commonly used inducing factors such as mitomycin C or UV light. Upon treatment with these factors, bacteria can be grown in a liquid medium until signs of lysis (if any) are observable. Lysate that has been treated with DNase can be used as a source of phages to prepare phage DNA for PCR amplification with prophage-specific primer pairs. The inducible factor-treated cells can also be streaked on a soft agar medium with suspended phage-sensitive cells (in a Petri dish). If the prophage was induced, the lysis zone in the underlying sensitive cell layer should surround each growing colony of lysogen. However, a limitation of the latter method is often the lack of a prophage-free strain able to serve as an indicator. 

## 15. Elimination of Prophages from Phage Propagation Strains

Traditional phage curing methods have been based on the selection of bacteria that have lost the prophage spontaneously or in response to inducing factors. If the prophage excision system is functional, prophage induction can be used to cure bacteria from that prophage [60]. Following prophage induction, cells are plated on a solid medium and tested for lysogeny. Prophage insertion in a chromosome may be associated with a specific phenotype, if it interrupts a gene of easily recognizable function. Curing from such prophages is associated with recovery of the wild-type strain phenotype, which may help to recognize prophage-free cells [62,94,141]. However, of the approximately 60% of phages that use intragenic regions as their attachment sites, over half have the attachment sites in tRNA encoding genes [105]. Additionally, other genes interrupted by prophages rarely have an easily recognizable phenotype. An additional difficulty may be “prophage jumping”—certain prophages excised from the primary attachment site can temporarily integrate into a secondary attachment site in the same cell, and thus the loss of phage conversion phenotype is not always associated with phage loss [94]. In such cases, the loss of prophage can be verified by testing cells’ sensitivity to a parental strain phage or by PCR with a prophage-specific primer pair. If factors that induce the excision and lytic development of a given prophage cannot be identified, one can search for colonies of spontaneously cured cells in a population of lysogens by plating lysogen culture cells onto a solid medium, growing them, and testing by colony blot for the presence of prophage [142]. An amplicon of any prophage-specific gene can serve as a probe in blotting tests. 

The overexpression of a cloned prophage excisionase gene in a respective lysogen can increase the frequency of prophage cured cell formation, as was shown in the case of lambda or KplE1 phage lysogens [143,144]. In certain cases, one prophage supports the excision of another prophage in the same cell by providing a helper function [145]. The removal of all active prophages from such cells using traditional methods is impossible. Thus, more reliable methods of prophage-free bacteria construction rely on recombineering techniques. For example, the *S. aureus* strain Newman was cured of four prophages by recombinational replacements of prophage-containing regions with the prophage-free regions of attachment sites for these phages cloned in temperature-sensitive replicon-based suicidal plasmids [146]. A curable plasmid expressing phage λ Red recombination system genes was used to replace four prophages in the *E. coli* chromosome with a PCR-amplified antibiotic resistance cassette, which was then eliminated with the help of another curable plasmid [128]. 

## 16. Future Possibilities to Produce Industrial Phage Propagation Strains 

The construction of new phage propagation hosts using traditional approaches might be a never-ending story possibly requiring hundreds of strains to be cured of plasmids, active prophages, and possibly other mobile genetic elements. However, taking into account recent achievements in synthetic biology as well as the progress in recombineering and genome editing methods, this need not be the case.

Whether a given phage infects a given bacterial strain from a susceptible species depends on the features of the bacterium and the phage. Metabolic compatibility of a bacterium with a phage to support the phage propagation in already-established infection appears to be species-specific, but sometimes it is extended to more than one bacterial species of the same or different genera [147,148]. Differential phage susceptibility determinants that are encoded by various strains of the same species include genes encoding phage receptors or pathways of their synthesis and phage-compatible restriction-modification systems [149,150,151,152,153,154,155]. Additionally, bacteria encode phage defence mechanisms, but these mechanisms protect the bacterium by itself either from infection with certain phages or from phage propagation, or induce apoptosis to protect the population from spread of the infection [156,157,158,159,160,161,162,163]. The differential phage susceptibility determinants are exchangeable between strains of a given species. Bacteria can gain or lose sensitivity to a given phage or the ability to support this phage development by mutation-, recombination-, or horizontal gene transfer-driven changes in their phage susceptibility or phage defence determinants [151,164,165,166,167,168,169,170,171,172,173,174,175]. Several genes associated with phage resistance or susceptibility are carried by mobile genetic elements [120,158,175,176,177,178,179,180,181,182,183,184,185,186,187]. 

Phage features important for the successful infection of a metabolically-compatible host include the compatibility of phage receptor binding proteins with receptors at the surface of a bacterial cell, the compatibility of phage genome modifications with the restriction-modification system of a bacterium, or the ability to prevent the action of bacterial restriction-modification systems either by avoiding sites that are recognized by the bacterial restriction-modification systems or by encoding efficient anti-restriction mechanisms [149,188]. Additionally, to productively infect bacteria, phages encode proteins that allow them to overcome bacterial phage resistance mechanisms, such as anti-CRISPR proteins and proteins that prevent the action of bacterial Abi or toxin–antitoxin (TA) systems [189,190]. 

The structure of each phage and its infectivity for particular hosts are determined by the genome of this phage. The only host-determined features of a phage seem to be certain epigenetic modifications, namely host-specific DNA methylation patterns [191,192]. They strongly influence the efficiency of infection of new hosts by a phage, being responsible for the limitations of horizontal gene transfer by bacteriophages [86,191,193,194]. Thus, in addition to species-specific basic metabolic pathways supporting the efficient propagation of a given phage, a phage propagation strain should be equipped with surface receptors for this phage attachment, cell envelope structures susceptible to the action of given phage lytic proteins, and a restriction-modification system that will allow the phage released from this strain to infect a desired set of clinical strains. The removal from such a strain of genetic determinants of other phage defence mechanisms (e.g., CRISPR/Cas, Abi, or TA loci), if any are encoded by its genome, could extend the number of phages able to propagate in its cells to phages infecting strains of the same species and using the same host receptors, but unable to overcome the respective phage-defence mechanisms. The acquisition of sensitivity to certain phages upon the abolishment of various bacterial phage defence systems has been demonstrated in several cases [120,195,196,197,198]. 

An optimal future strategy to acquire therapeutic phage propagation strains of desired properties may be the construction of a bacterial chassis of selected clinically relevant pathogenic species. In synthetic biology, a chassis refers to the organism serving as a foundation to physically house genetic components and support them by providing the resources for basic functions, such as replication, transcription, and translation machinery [199]. The bacterial chassis strains to serve as basic platforms for the construction of industrial phage propagation strains should have genomes reduced in their complexity and the content of undesired genes by the depletion of most of the mobile genetic elements as well as virulence and phage resistance determinants—a procedure that is known as a top-down strategy of the genome reduction process [200]. Additionally, they should be ready for the introduction or exchange of genomic modules (e.g., an appropriate restriction-modification system or phage receptors determining gene cassettes), enabling these strains to serve as microbial cell factories for the propagation of selected therapeutic phages. Methodologies enabling the abolishment of mobile genetic elements and other genome fragments using genome shuffling, recombineering, oligo-mediated allelic replacement, or genome editing using CRISPR/Cas-assisted selection of desired clones have been developed for model bacteria, even on a genome-wide scale [201,202,203,204,205,206,207,208,209]. The repertoire of genetic engineering tools that extend the ability of genomic manipulations to bacteria other than *E. coli* using the newest strategies has been constantly increasing, providing means to edit genomes belonging to genera represented by the most problematic bacterial pathogens, including potential phage propagation strains [210,211,212,213,214,215,216,217,218].

The results of studies on bacteria that were cured of some or most of the recombinogenic or mobile genetic elements (including prophages) indicate that they have several advantages. For instance, *Escherichia coli* K-12 with a genome reduced by 15% by the removal of mobile DNA and cryptic virulence genes preserved good growth profiles and protein production as well as the accurate propagation of recombinant genes and plasmids that could not be stably propagated in other strains [219]. The growth properties and endurance of environmental stresses of a *Pseudomonas putida* KT2440 derivative which was cured of prophages, some transposons, and some restriction-modification cassettes was found to be superior to its wild-type parent [220,221]. Curing a *Corynebacterium glutamicum* industrial strain of prophages caused an increase of strain fitness, stress tolerance, transformability, and protein production yield [222]. Thus, in our opinion, the construction for the propagation of therapeutic phages, of chassis strains equipped with certain phage susceptibility determinants and depleted of phage resistance determinants as well as certain mobile genetic elements or virulence determinants will not only ensure the safety of therapeutic phage preparations, but will also reduce the cost of phage production substantially. This reduction will be a result of: (i) minimizing the number of strains required for the production of different phages; (ii) eliminating the need of evaluating phage preparations for the content of undesired elements, including temperate phages and toxins; and (iii) increasing the fitness and stability of such strains in the industrial production of therapeutic phages. Additionally, one foundation strain constructed for a bacterial species can serve as a platform for the enrichment of its genome with various gene cassettes required for the propagation of various phages. We have already constructed basic prophage- or plasmid-free strains to start the development of a chassis of *S. aureus* and *E. faecalis* strains. They serve for the production of monoclonal preparations of certain *S. aureus* and *E. faecalis* phages [62,223]. Further work to remove additional undesired genomic elements from the genomes of these strains is in progress. 

## 17. Surrogate Hosts for the Propagation of Therapeutic Phages

The use of non-pathogenic relatives of pathogenic strains enabling therapeutic phage propagation was proposed to eliminate the problem of phage preparations’ contaminants derived from virulent phage propagation hosts [224,225]. Unfortunately, suitable “surrogate” hosts can be found only in a limited number of cases, and not all of them enable the efficient propagation of therapeutic phages [226,227,228,229,230,231]. Additionally, long-term effects of the enrichment of a pathogenic strain population with prophages released from strains believed to be non-pathogenic are impossible to predict, especially in view of documented cases of infections caused by certain strains belonging to the surrogate host species [232,233,234,235,236,237,238,239] and cross-species transfer of mobile genetic elements between representatives of surrogate host species and their pathogenic relatives [240,241,242,243,244,245,246]. Moreover, genomic analysis of pathogenic strains of certain species and their relatives representing non-pathogenic species indicates that the latter may function as reservoirs of accessory genes for the former [103]. Thus, even when using surrogate non-pathogenic hosts for the propagation of therapeutic phages, the removal of prophages from such hosts may be a wise strategy to avoid unpredicted problems in the future.

## 18. Economic Aspects of the Industrial Construction of Phage Propagation Strains

In nature, prophages are temporary components of bacterial genomes which can enter, exit, or change their location in the genome. Their loss is a natural process that occurs with various frequencies, as long as the mobility of a prophage is not abolished by deletions or other rearrangements that make the prophage remnants a permanent part of the genome. Thus, in most cases, the major cost of acquiring cells that are depleted of active prophages is the cost of screening (labour, media, and blotting or PCR reactions), and sometimes the cost of recombineering and genome editing techniques, provided the availability of tools. Economic aspects argue for going further and constructing species-specific bacterial chassis for the production of therapeutic phages by the removal of plasmids, if any, and chromosomal elements that cause genome mutability, phage resistance, or encode virulence factors. The construction of such strains could be done based on recombineering and genome editing methods analogous to those that have been used in the process of modification of bacterial producers of various compounds for industry [89,222,247,248,249,250,251,252,253,254,255]. Subsequently, such a chassis strain could be used as a platform for the exchange of particular phage-sensitivity determinants in its genome with selected strains sensitive to certain phages. The economic benefits of such an approach would be associated not only with the increased safety of phage preparations produced with the use of these strains, but also with a switch from many different strains of various properties to fewer strains of the same core genome and only a few gene cassettes to be exchanged. Results of studies on certain model or industrially-applicable bacteria that were depleted of prophages and certain other mobile elements as well as certain determinants of mutability indicate that such strains have a better genomic stability and are more efficient producers of certain compounds than their wild-type parents [89,199,219,252,254,255]. Engineering of their genomes does not need to be associated with the permanent presence of heterologous DNA, as markerless gene knock-out or gene replacement systems have been developed for a number of pathogenic bacterial species and are in constant further development [254,255,256,257,258,259,260,261,262,263,264,265,266,267,268,269,270,271,272,273,274,275,276].

## 19. PT: Beyond the Antibacterial Action

In recent years, data have been accumulating indicating that phages may also interact with mammalian cells, thus “crossing the border to eukaryotic cells”—binding to their surface receptors and penetrating into them. Phages can therefore pass across confluent epithelial cell layers and migrate to blood, lymph, and other tissues [51]. These findings essentially confirm our hypothesis of “phage translocation” from the intestines [277] extended by Barr, who used the term “journey” to suggest that phages travel through the human body [278]. Phages have been shown to mediate anti-inflammatory and immunomodulating properties [279]; therefore, such phenomena may be relevant for the maintenance of immunological homeostasis. Consequently, we recently hypothesized that phage therapy may be considered for treating disorders such as inflammatory bowel disease, autoimmune hepatitis, allergy, as well as some viral infections [280,281,282,283]. Evidently, this requires further work and confirmation by relevant clinical trials. While the most trustworthy advances come through the performance of well-designed trials, sometimes experimental treatments based on theoretical considerations alone may lead to major breakthroughs [284]. As stated, “the potential for broader application of phage therapy is evident and it is certainly worthy of further studies” [285].

## 20. Conclusions

Almost a century after its consolidation in Eastern countries, a silver lining is appearing on the horizon for phage therapy in the Western world. The increased threat of antibiotic resistance makes all stakeholders in the sector of infectious disease feel a high pressure to find new antibiotics and search for safe alternatives. In this situation, phage therapy is increasingly considered as a potential alternative or auxiliary tool. More and more patients, doctors, pharmacists, media, authorities, and industry show their active interest and signs of a more open mind to assess the possible benefits of phage therapy. This is especially triggered by an increasing number of publications of patient cases where spectacular results were achieved with bacteriophages. It is now essential that the efficacy and safety of phage application be demonstrated in rigorous clinical trials. National and international authorities are opening their doors to such trials, and are prone to regulate phage therapy if it is found to be effective and safe. Furthermore, progress in research on phage biology suggests that other applications of phages unrelated to their anti-bacterial action may be on the horizon.
